# Motivations and expectations of pregnant women using psychoactive substances during prenatal care: phenomenological study

**DOI:** 10.17533/udea.iee.v42n2e10

**Published:** 2024-07-08

**Authors:** Júlia Oliveira Silveira, Mara Regina Caino Teixeira Marchiori, Andressa da Silveira, Fabiana Porto da Silva, Zaira Letícia Tisott, Kelvin Leandro Marques Monçalves, Keity Laís Siepmann Soccol

**Affiliations:** 1 Nurse. Health Service of Restinga Seca, Brazil. Email: oliveirasilveiraj2@outlook.com Prefeitura Municipal de Restinga Seca Health Service of Restinga Seca Brazil oliveirasilveiraj2@outlook.com; 2 Nurse, PhD. Professor, Professional Master's Degree in Maternal and Child Health. Email: maramarc@ufn.edu.br Universidade Federal de Santa Maria Brazil maramarc@ufn.edu.br; 3 Nurse, PhD. Professor, Nurse College of the Federal University of Santa Maria. Palmeira das Missões, Brazil. Email: andressa-da-silveira@ufsm.br. Universidade Federal de Santa Maria Nurse College of the Federal University of Santa Maria Palmeira das Missões Brazil andressa-da-silveira@ufsm.br; 4 Nurse, Master. Professor, Nursing College. Email: fabiana.silva@ufn.edu.br Universidade Federal de Santa Maria Nursing College Brazil fabiana.silva@ufn.edu.br; 5 Nurse, PhD. Porto Alegre, Brazil Email: zairatisott10@gmail.com Porto Alegre Brazil zairatisott10@gmail.com; 6 Nurse, Master student. Email: kelvinmmoncalves@hotmail.com kelvinmmoncalves@hotmail.com; 7 Nurse, PhD. Professor, Professional Master in Maternal and Child Health. Email: keitylais@hotmail.com. Corresponding author. Universidade Federal de Santa Maria Brazil keitylais@hotmail.com; 8 Franciscana University. Santa Maria, Brazil Universidade Federal de Santa Maria Franciscana University Santa Maria Brazil

**Keywords:** substance-related disorders, pregnancy, prenatal care, maternal and child health, primary health care, trastornos relacionados con sustancias, embarazo, atención prenatal, salud materno-infantil, atención primaria de salud., transtornos relacionados ao uso de substâncias, gravidez, cuidado pré-natal, saúde materno-infantil, atenção primária à saúde.

## Abstract

**Objective.:**

Understand the motivations and expectations of pregnant women using psychoactive substances during prenatal care.

**Methods.:**

A qualitative study developed in the light of Alfred Schütz's Theoretical Framework of Phenomenological Sociology, in which 25 pregnant women using psychoactive substances, belonging to a Family Health Strategy, participated. Data production took place between August and November 2022.

**Results.:**

Two units of meanings emerged: (i) social influences for the performance of prenatal care and (ii) expectation regarding the care to be received by the health professional. Pregnant women do pre-natal due to family influences, for fear of losing their children due to loss of guardianship and concern about the well-being and development of the baby. And, the expectations are that they receive good attention, feel safe when they are attended to by health professionals and also that they are understood and have a relationship of trust.

**Conclusion.:**

Pregnant women who use psychoactive substances bring motivations for prenatal care linked to the past, such as influences from family members and previous experiences. As for expectations, they are related to the child's health and the care expected by professionals. Finally, strategies to reduce harm during pregnancy of users of psychoactive substances are fundamental for the effectiveness of care.

## Resumen

**Objetivo.:**

Conocer las motivaciones y expectativas de las gestantes consumidoras de sustancias psicoactivas durante la atención prenatal.

**Método.:**

Se trata de un estudio cualitativo basado en el marco teórico de la sociología fenomenológica de Alfred Schütz, en el que participaron 25 gestantes consumidoras de sustancias psicoactivas pertenecientes a una Estrategia de Salud Familiar de una unidad de salud en Brasil. Los datos se recogieron entre agosto y noviembre de 2022.

**Resultados.:**

Emergieron dos unidades de significado: (i) influencias sociales para la atención prenatal y (ii) expectativas sobre la atención recibida por el profesional de salud. Las gestantes acuden al control prenatal por influencias familiares, miedo a separarse de sus hijos por pérdida de la tutela y preocupación por el bienestar y desarrollo del bebé. Sus expectativas son recibir una buena atención, sentirse seguras con los profesionales sanitarios y ser comprendidas y escuchadas a través de una relación de confianza**.**

**Conclusión.:**

Las embarazadas usuarias de sustancias psicoactivas tienen motivaciones para el cuidado prenatal relacionadas con su pasado, como influencias familiares y experiencias individuales previas. En cuanto a las expectativas, están relacionadas con la salud del niño y los cuidados esperados que les brindarán los profesionales de salud. Por último, el diseño de estrategias para reducir los daños durante el embarazo de las consumidoras de sustancias psicoactivas es fundamental para un cuidado eficaz.

## Resumo

**Objetivo.:**

Compreender as motivações e expectativas de gestantes usuárias de substâncias psicoativas durante a realização do pré-natal.

**Métodos.:**

Estudo de abordagem qualitativa desenvolvida à luz do referencial Teórico da Sociologia fenomenológica de Alfred Schütz, em que participaram 25 gestantes usuárias de substâncias psicoativas, pertencentes a uma Estratégia Saúde da Família. A produção de dados ocorreu entre os meses de agosto a novembro de 2022.

**Resultados.:**

Emergiram duas unidades de significados: (i) influências sociais para a realização do pré-natal e (ii) expectativa em relação ao cuidado a ser recebido pelo profissional de saúde. As gestantes realizam o pré-natal por influências de familiares, por receio de afastamento dos filhos decorrente de perda da tutela e pela preocupação com o bem-estar e desenvolvimento do bebê. E, as expectativas é de que recebam um bom atendimento, sintam segurança nos profissionais de saúde e que sejam compreendidas e ouvidas por meio de uma relação de confiança.

**Conclusão.:**

As gestantes usuárias de substâncias psicoativas trazem motivações para a realização do pré-natal atreladas ao passado, como influências de familiares e experiências prévias. Quanto as expectativas, estão relacionadas à saúde da criança e o cuidado esperado pelos profissionais. Por fim, as estratégias para reduzir danos durante a gestação de usuárias de substâncias psicoativas é fundamental para a efetividade do cuidado.

## Introduction

The pregnancy period is characterized by being a different experience in women's lives, because at that moment there are numerous changes, from physiological, psychological and even social, which requires actions aimed at adequate and quality care for the pregnant woman.^1^ In view of these changes, prenatal care is fundamental to boost health promotion, as it aims to promote care for women, taking into account all the singularities that involve this process.^2^However, although prenatal care is fundamental for the care of pregnant women, there are still difficulties in accessing health services, especially for women living in vulnerable situations,^2^ among them, women who use psychoactive substances (PAS) stand out. Also, the factors that predispose pregnant women who use PAS should be considered. These women are in contexts of vulnerability due to issues of gender, race, education, abusive family relationships, addictive behavior in the family and violence.^3^

It is estimated that about 20% of women use some type of PAS during pregnancy.^4^However, there is still an underestimation of the prevalence of this population,^5^ which indicates that this data may be higher. The prevalence of prenatal PAS use is difficult to estimate due to gaps in identification.^6^In addition, rates of PAS use continue to rise among this population.^7^^,^^8^ There is a worldwide concern about the use of PAS by pregnant women, so it is already considered an important public health problem that affects the health of mother and baby.^6^^,^^9^^,^^10^ The use of PAS, whether licit or illicit, causes negative outcomes for maternal and child health,^6^^,^^7^ causing spontaneous abortions, premature birth, low birth weight and congenital malformations. 

In view of this, the importance of a prenatal care that meets the uniqueness of pregnant women using a PAS is emphasized, given that when pregnant women use a PAS, they experience prejudice and stigmas even by health professionals. Prenatal care for pregnant women using PAS needs to be differentiated, considering that drug users deny that they use such drugs or are dependent on them; because of this, these women do not seek prenatal consultations and health care, or when they seek this help it is already too late.^3^

The theme of pregnant women using PAS has many issues to be discussed and unveiled, especially those related to prenatal care, in order to drive improvements in care and understanding of the life context of these pregnant women. Thus, giving voice to pregnant women and understanding the uniqueness, experiences and social relations for prenatal care are fundamental. Given the above, this study aimed to understand the motivations and expectations of pregnant women using PAS during prenatal care. 

## Methods

Qualitative approach study developed in the light of Alfred Schütz's Theoretical Framework of Phenomenological Sociology. ^11^ Phenomenological sociology allows the understanding of the meaning of the actions, experiences and social relations that human beings experience in the world of life, as well as interpreting their experiences. According to the framework, when women act in the world thinking about something they have already experienced or experiences they have observed in their social relationships, they reveal the reasons "why". And, when they act with prospects for the future, they express their intentionality, thus revealing the "reasons for". 

The study was developed in a Family Health Strategy (FHS) of a municipality located in the central region of southern Brazil, with pregnant women using PAS who performed prenatal care at the service. The inclusion criteria for this study were: being pregnant and using psychoactive substances, prenatal care with a physician or nurse in the FHS during the data collection period, and being 18 years of age or older. As exclusion criteria, being under the effect of a PAS during the prenatal consultation or interview, or presenting problems for communication. The setting and data collection took place between August and November 2022, by the main researcher of the study, female, who was a student of the undergraduate nursing course. The setting and approach to pregnant women occurred prior to the interviews, in which the researcher had already participated in health education groups with pregnant women in the aforementioned service. In addition, the student completed the final stage of the nursing course, with a high semester workload in this place, which made the pregnant women get to know her and feel comfortable talking to her, as well as sharing prenatal consultations with the nurse or physician of that service. 

The student had the assumption that pregnant women did not do prenatal care only due to the use of drugs, which sometimes made it impossible for them to go to the service, but that the approach of health professionals interfered with their care and reception. The student's interest in developing the research is related to her trajectory, in which she observed, in different health services in the city during the course's practical classes, the weaknesses in assistance and social exclusion that pregnant women who use drugs experience in their daily lives.

Data collection took place through phenomenological interviews, which provided an opportunity to understand how pregnant women express their lived experiences and expectations in relation to prenatal care and the care received by the health team. In this sense, the approach and setting contributed significantly to data collection, as it was necessary to establish bonding and empathy relationships so that pregnant women felt comfortable to express their experiences during the phenomenological interview. Thus, the interviews were conducted by the main researcher. The student was trained to conduct phenomenological interviews. The training was given by the PhD professor and researcher responsible for the research, who has experience in phenomenological research. For data collection, the researcher followed a predefined script with the characterization of the pregnant women, which contained age, education, profession or occupation, history of use and type of PAS used by a family member, and type of PAS she used. Together with the characterization questions, the following questions were asked: "What are the reasons that lead you to do prenatal care?" "What are your expectations when performing prenatal care?" "What do you expect from health professionals when performing prenatal care?" 

Interviews were scheduled in advance, by verbal and face-to-face invitation, shortly after prenatal consultations or group health education activities. The invitation to participate in the interview was intentional. There were no refusals to participate in the survey. These were carried out individually, in which only the pregnant woman and the researcher were present in a room in the health service, in order to maintain the privacy of the women and the confidentiality of the information. A digital recorder was used to capture the participants' utterances, and the recordings lasted a mean of 35 minutes per interview. The interviews ended when the sufficiency of meanings was reached, that is, when the information began to repeat itself and no new information emerged.^12^

The testimonies were transcribed in full in the Microsoft Word Software by the main researcher, concomitantly with the collection period. The conference was held based on attentive listening to the interviews and dynamic reading of the transcripts by the main researcher and the responsible PhD professor, in order to ensure the reliability of the information and the veracity of the data. For the analysis, the paths elaborated by researchers of phenomenological sociology were used,^13^ where the speeches were read and reread in text form, in order to understand the reasons and expectations of pregnant women in relation to prenatal care. Therefore, excerpts were identified through chromatic coding and selected speeches referring to the proposed objectives. After selecting these data, the units of meanings were identified and grouped according to their similarity, which allowed the construction of the concrete categories of the lived experience. 

The interpretation of the results was analyzed through the theoretical conceptions of Alfred Schütz's Phenomenological Sociology and related literature. During all phases of the preparation of this study, the ethical principles were followed, as provided for in Resolution number 466, of December 12, 2012, of the National Health Council and Resolution number 510, of April 7, 2016, of the National Health Council/ Ministry of Health (CNS/MH) directed to research with human beings. All participants signed the Informed Consent Form. The study was approved by the Research Ethics Committee of the Franciscana University under opinion number 5.183.201 on December 21, 2021. 

## Results

The study included 25 pregnant women, aged between 20 and 41 years, with a history of previous pregnancies. Of these, 20 had a family history of drug use, by parents, siblings or current spouse/partner. Regarding education, 3 women studied until complete high school (CHS), 4 had completed elementary school in Youth and Adult Education (EJA), while 18 studied until elementary school (ES). With regard to the profession, 10 had occupations as housewives, 7 worked in local business and 8 were unemployed. 

The profile of women denotes that they are mostly away from the labor market even at a productive age and that their education predominates in ES. However, it cannot be said that these conditions are due to the excessive use of PAS that can disable them and hinder the work and study routine. Economic conditions, income, access to education and job opportunities were not discussed in this study. As for the type of drug, pregnant women are users of multiple drugs such as tobacco, cocaine, crack, marijuana and alcoholic beverages (Table 1). From the analysis of the phenomenological interviews, two concrete categories of the experience were revealed: Social influences for the performance of prenatal care and Expectations regarding the care to be received by the health professionals. 

**Table 1:** Characteristics of the 25 pregnant women participating in the study

Code	Age (years)	Education	Occupation	Previous pregnancies	Family history and type of PAS	Type of PAS you use
PW1	25	CHS	Trade store seller	2	Mother: tobacco Husband: tobacco & Alcoholic Beverages	Alcoholic beverages, tobacco and marijuana
PW2	27	ES	Local business: autonomous seller	6	Mother: uses tobacco Father and Husband: Alcoholic Beverages	Alcoholic beverages and tobacco
PW3	32	YAE	Housewife	1	Mother, father, brothers and sisters-users of tobacco and alcoholic beverages	Alcoholic beverages, tobacco and marijuana
PW4	30	CHS	Unemployed	3	Husband: tobacco, Alcoholic beverages and crack	Alcoholic beverages
PW5	39	ES	Housewife	5	Husband: tobacco and Alcoholic Beverages	Alcoholic beverages, tobacco and marijuana
PW6	41	ES	Housewife	3	Sister: marijuana and alcoholic beverages	Alcohol beverages, tobacco and cocaine
PW7	21	ES	Trade In-store seller	2	Mother: Alcoholic beverages Partner: Alcoholic beverages	Tobacco and alcoholic beverages
PW8	20	ES	Unemployed	1	Father: Alcoholic beverages Partner: cocaine	Alcoholic beverages, tobacco, marijuana, and cocaine.
PW9	25	ES	Housewife	1	None	Tobacco
PW10	20	ES	Trade self-employed candy seller	2	Mother, Brother and Husband: tobacco and alcoholic beverages	Alcoholic beverages and tobacco
PW11	25	YAE	Trade: saleswoman	3	Mother: tobacco Sisters: tobacco, alcoholic beverages and cocaine	Alcoholic beverages and tobacco
PW12	38	ES	Housewife	2	Father: tobacco and alcoholic beverages	Alcoholic beverages; and tobacco
PW13	34	ES	Unemployed	2	Husband: alcohol, cocaine and crack; Father: Alcoholic beverages	Alcoholic beverages, cocaine and crack
PW14	26	ES	Unemployed	1	None	Alcoholic beverages and crack
PW15	28	CHS	Unemployed	2	Partner: Alcoholic beverages and tobacco	Alcoholic beverages, cocaine and crack
PW16	27	ES	Housewife	2	None	Alcoholic beverages, tobacco and cocaine
PW17	23	ES	Housewife	1	Mother: tobacco and alcoholic beverages Partner: alcoholic beverages and cocaine	Alcoholic beverages, cocaine and tobacco
PW18	38	ES	Local business: cleaning of environments	3	None	Alcoholic beverages; and tobacco
PW19	32	YAE	Unemployed	2	Mother and brother: alcoholic beverages	Alcoholic beverages and cocaine
PW20	27	ES	Unemployed	2	Father: alcoholic beverages	Alcoholic beverages and marijuana
PW21	27	ES	Local business: informal older adults caregiver	1	None	Alcoholic beverages, tobacco and crack
PW22	33	ES	Housewife	3	None	Alcoholic beverages and cocaine
PW23	28	ES	Housewife	2	Sister: marijuana and tobacco	Alcoholic beverages, cocaine and crack
PW24	39	YAE	Housewife	2	Brothers: Alcoholic beverages Partner: tobacco	Alcoholic beverages and tobacco
PW25	29	ES	Unemployed	1	Mother: uses tobacco and alcoholic beverages Husband: alcoholic beverages	Alcoholic beverages, Cocaine and crack

### Social influences for prenatal care

This category shows the "reasons why" represented by the social influences that the pregnant women had, that is, it was a learning experience they had with their families. In this sense, the influence of the mother and sister for the performance of prenatal care is revealed, in which their experience made them learn that it is necessary to perform prenatal care. Thus, they reproduce the actions seized. In addition, they learned that this implies the possibility of losing custody of their children due to negligence: *I can't say, because by then I had already grown up with this learning from my mother. My [mother] always did prenatal care to see if the baby was okay (G1); I learned from my mother, who always said that she has to do prenatal care, that it is important to control, take care (G7); My sisters were all upset at the hospital, they almost lost their children! When you get there, they ask if you had prenatal care, if everything is registered on your card, because it seems like we are negligent with the baby if we don't do prenatal care (G11).*

The life history of the pregnant women, through their previous experiences, expressed by previous hospitalizations, threats of abortions and diseases caused in the gestational period, made the women perform prenatal care in the current pregnancy. Given the biographical situation, pregnant women do not have the same experiences, and therefore perform prenatal care: *The right thing is to do prenatal care! I learned when I was pregnant with my second child, because I didn't have prenatal care for my first daughter. I lived in the country and didn't know about prenatal care. And when I went to the hospital they told me that I had to have done it. I was admitted to the hospital, I had a threatened miscarriage; I think it was because I used [PAS] a lot. So, from that I learned that you have to do prenatal care (G6); The other time I was pregnant I didn't take prenatal care and I didn't take proper care of myself, because I was using a lot of drugs. Then my daughter was born with a problem. I had already heard about prenatal care, but I didn't do it because I was using [PAS]; I couldn't even think about anything at that time (G15).*

In addition, the loss of guardianship of the children due to the use of PAS, combined with the non-performance of prenatal care in previous pregnancies, caused the pregnant women to seek the health service to perform prenatal care. Thus, the action of seeking prenatal care is motivated by not having to go through the removal of the children again: *I lost custody of my son. I was using drugs all the time. And then, I didn't come for prenatal care either. And, as I wasn't there for follow-up, they contacted the Guardianship Council. I lost my son because of myself! (G14); I had a lot of problems with the Child Protection Council, because they took custody of my son, my mother is the one who has custody. They said that I didn't take care of the baby, that I was negligent. I didn't do prenatal care. And I was still using a lot of drugs at that time. I had my son but I wasn't a mother. I couldn't be a mother to my own child (G15).*

In addition, when performing prenatal care they have expectations, represented by the "reasons for", in which they are motivated by the possibility of sharing their life with their successors and avoiding the status of being negligent before the Guardianship Council: *This son here, I don't want custody taken away from me (G14); Because if I didn't get prenatal care I would lose my baby! Because otherwise, when it was time to have the baby, they would bother you. They said they would call the Guardianship Council to take my baby away (G2); You have to do prenatal care nowadays, because if you don't do it, the Guardianship Council will come after you. They call the council because they say we are neglecting our son. Because we miss the appointment, we don't take the exams, we don't receive the vaccines (G10); It's so we don't get upset in the hospital. Otherwise, they will contact the Guardianship Council. And they can even take our child away from us (G11).*

Still in the case of social influences, pregnant women when performing prenatal care have expectations directed to the health of their babies. In this sense, the "reasons for" are linked to the monitoring of the baby's growth and development, as well as the concern that the baby may be born with some damage resulting from their use of PAS: *If the little heart is beating normally, and everything is going well. And to be born healthy. I'm afraid that the fact that I use drugs will interfere with something (G1); To see the baby's health. Whether it will be born well or not, because I'm afraid because of the things I use [psychoactive substance] (G5); I'm afraid the baby will be born with problems, so I do the exams, I come to the appointments correctly. I think it's possible to follow it better, to know what's going on inside the belly (G19).*

### Expectations regarding the care to be received by the healthcare professional

The expectations that pregnant women have in relation to the care of the health professionals who accompany them during the prenatal consultation, is that they receive good care, have guidance about their health and the baby, so that they feel safe in the pregnant-professional relationship, through a clear and understandable dialogue: *We expect to be treated well. Today was a day that paid attention to me. They did everything they were supposed to do. I'm not leaving with the pain I arrived with. I hope they examine it, talk to us (G3); I wanted them to explain it well, because nobody actually told me what pre-eclampsia was. I didn't even know what that was! I found out when I felt ill and went to the hospital, and then they said I could die, either me or my daughter (G7); I hope they guide the right things for us (G11).* Furthermore, they expect health professionals to break the relationship of disbelief in the face of statements about having stopped using PAS. Thus, they expect care to be based on the establishment of a relationship of trust: *I used drugs in previous [pregnancies], which I used a lot. And no matter how much I say that I stopped using it, they don't believe it. Now it's just drinking, and not much, like it was before (G2); And the nurse said: “Are you sure you’re not smoking anymore?” I said, “I'm sure! I'm not smoking anymore. I only drink a few beers from time to time, but I no longer drink other strong drinks” (G4).*

Pregnant women have knowledge about the losses of using a PAS and the act of stopping using it is something that becomes difficult in their daily lives. In this sense, when they want to be understood by professionals, they reveal the "reasons for": *They do everything they can for the baby's sake, they pay attention to me during the consultation, but they always say that I need to stop smoking and drinking because it's harming the baby. I feel charged, but I can't stop (G12); “I wanted them to understand my whole situation. I've been using it for years and I know it's bad for children, and yet the desire to use it is greater. I couldn't stop using it. And they charge me a lot of it! I find this pressure annoying, I feel low, it's like I don't want to take care of the baby (G17); I've cut down on marijuana a lot, but I still smoke. And I only drink on the weekends, but I haven't stopped. And in consultations they always ask and say to stop. If it were easy, I would have stopped already. They need to understand that it's not like that, that it's not a button that I press and then I stop (G20); I slowed down, but drugs rule us! I feel like a total failure. And I'm very afraid that my daughter will be born with a problem because of me (G25).*

The statements reveal that pregnant women are aware of the harm caused by drug use during pregnancy, but the addiction remains, even with the reduction in consumption. The participants also praised the guidance received and their feelings regarding the guidance to stop drug use.

## Discussion

The findings of this study reveal that the motivation to perform prenatal care is related to the influences that pregnant women had from their mother and sister, so prenatal care is part of the culture of these family nuclei. The learning that pregnant women obtained, through the establishment of social relationships and sharing of experiences boosted them to adhere to prenatal care. In this sense, the act of performing prenatal care was learned from peers, because the world in which people live and exchange occurs is intersubjective and cultural. Thus, prenatal care proved to be a socially constructed custom,^11^ in which the family assumes a role of social cohesion.^14^Another reason that led pregnant women to undergo prenatal care is related to previous experiences of previous gestational diseases, which culminated in hospitalization, and also because they experienced threats of abortion. This can be corroborated in a study that shows that pregnancy will not always be a moment permeated only with happiness, pleasure and positivity, since it is a subjective experience and each pregnant woman will experience it from her point of view.^15^

The experiences of pregnant women in the world of life, together with their biographical situation, were present as motivations for prenatal care. Pregnant women act in the world of life according to the knowledge they have acquired in their life trajectory. Thus, their stories are constituted by subjective experiences.^11^Among the experiences they had, the loss of guardianship of their children was also a motivation for prenatal care. The loss of the right to live with their children left marks on the memory of pregnant women, causing them feelings of suffering and distress. The biographical situation is unique to each person, and brings with it the memories and marks of the past.^11^In this sense, they do prenatal care in order not to have to go through the same life experiences.

Also, they have the intention of monitoring the intrauterine development of the baby, and perceive prenatal care as something relevant and as a possibility to know if the baby is developing well. They demonstrate that they are aware that the use of psychoactive substances affects the development of the baby, as already evidenced in the literature,^16^^,^^17^ but they cannot stop using it. As well as, the imposition of ceasing use impairs the establishment of the intersubjective relationship between the professionals of the pregnant women, which has repercussions on the rupture of the relationship-of-us.^11^The use of drugs by women by themselves is permeated by stigmatization and as something inappropriate, which causes them to be judged morally.^18^

Pregnant women want good care, adequate guidance, through a clear dialogue and that the relationship established makes them feel safe and is based on trust. Still, they expect to be understood by professionals, because the relationship of subordination and distrust causes them to be demotivated to perform prenatal care. Therefore, it is important to establish a relationship of trust, since many women do not verbalize or partially report the use of PAS due to the discomfort that occurs during screening and which may also be related to the reduced workload of professionals, which causes consultations to occur in a limited time.^19^Some pregnant women mention the discomfort they feel when professionals act in a prohibitionist way about the use of PAS, ignoring their subjectivity and their mental health. This type of relationship leads to abandonment of prenatal care, as people live in a network of complex social relationships and expect to have their expectations met in relation to the other.^11^Also, the need for singular care for pregnant women was revealed, taking into account the paradigm of harm reduction and not only abstinence. For this, it is important to strengthen measures and develop new strategies aimed at understanding, empowering and choosing women's habits during pregnancy.^20^

Professionals should develop their ethical and non-exclusive practices, and use harm reduction strategies.^21^The prenatal care of women who use PAS is marked by unsatisfactory reception practices, insufficient health educational information and fragile ties with the health team.^3^Other ways of caring are needed, from the perspective of human rights and harm reduction, so that assistance does not translate into violence against women and their withdrawal from services.^(3.22)^ Identifying the factors that are associated with the use of PAS during pregnancy is essential for the identification and treatment of pregnant women to reduce risks and to improve outcomes for maternal and child health.^8^The low access of pregnant women using PAS to prenatal care culminates in a greater risk of obstetric and fetal complications,^4^ which even has repercussions on leave in the postpartum period.^23^

Low adherence to services is a response associated with psychosocial and sociodemographic crossings^24^ and lack of reception and bonding with prenatal health teams.^3^Therefore, there is a need for the development of continuing education for professionals in order to improve the understanding of aspects related to mental health and social and cultural differences. The study allowed us to advance in the understanding of the life contexts, motivations and expectations of pregnant women who use PAS and who perform prenatal care. The (re)knowledge of these contexts in the gestational period can be useful in the formulation of harm reduction strategies and health problems related to the use of PAS during pregnancy, leading to a favorable gestational outcome and a humanized care based on the uniqueness and biographical situation of each pregnant woman.

This study allowed us to elucidate the motivations and expectations of pregnant women using PAS during prenatal care and understand them through the sociological phenomenology of Alfred Schütz. During prenatal care, pregnant women refer to the past to bring the reasons for carrying out care during pregnancy as family influences and previous experiences, but also to the future with expectations related to the baby's health, combined with the care expected by professionals in the sense of attention, guidance and understanding about the use of PAS during pregnancy. Understanding the motivations and expectations of pregnant women allows the construction of comprehensive care from the perspective of reducing damage to the health of the pregnant woman and the baby, as well as for the construction of specific public policies for this population. Finally, strategies to reduce harm during pregnancy of users of psychoactive substances are fundamental for the effectiveness of care. 

The limitation of the study is related to the profile of pregnant women using PAS in a single health territory, since it is necessary to further deepen the motivations and expectations of pregnant women in a comprehensive way, however it highlights a reality that can occur in several scenarios.
